# Inactivation of SARS-CoV-2 in All Blood Components Using Amotosalen/Ultraviolet A Light and Amustaline/Glutathione Pathogen Reduction Technologies

**DOI:** 10.3390/pathogens11050521

**Published:** 2022-04-28

**Authors:** Felicia Santa Maria, Yan-Jang S. Huang, Dana L. Vanlandingham, Peter Bringmann

**Affiliations:** 1 Cerus Corporation, Concord, CA 94520, USA; fsantamaria@cerus.com; 2 Department of Diagnostic Medicine/Pathobiology, Biosecurity Research Institute, Kansas State University, Manhattan, KS 66506, USA; yshuang1985@bri.ksu.edu (Y.-J.S.H.); dlvanlan@bri.ksu.edu (D.L.V.)

**Keywords:** SARS-CoV-2, pathogen reduction technology, amotosalen, amustaline

## Abstract

No cases of severe acute respiratory syndrome coronavirus 2 (SARS-CoV-2) transfusion-transmitted infections (TTI) have been reported. The detection of viral RNA in peripheral blood from infected patients and blood components from infected asymptomatic blood donors is, however, concerning. This study investigated the efficacy of the amotosalen/UVA light (A/UVA) and amustaline (S-303)/glutathione (GSH) pathogen reduction technologies (PRT) to inactivate SARS-CoV-2 in plasma and platelet concentrates (PC), or red blood cells (RBC), respectively. Plasma, PC prepared in platelet additive solution (PC-PAS) or 100% plasma (PC-100), and RBC prepared in AS-1 additive solution were spiked with SARS-CoV-2 and PR treated. Infectious viral titers were determined by plaque assay and log reduction factors (LRF) were determined by comparing titers before and after treatment. PR treatment of SARS-CoV-2-contaminated blood components resulted in inactivation of the infectious virus to the limit of detection with A/UVA LRF of >3.3 for plasma, >3.2 for PC-PAS-plasma, and >3.5 for PC-plasma and S-303/GSH LRF > 4.2 for RBC. These data confirm the susceptibility of coronaviruses, including SARS-CoV-2 to A/UVA treatment. This study demonstrates the effectiveness of the S-303/GSH treatment to inactivate SARS-CoV-2, and that PRT can reduce the risk of SARS-CoV-2 TTI in all blood components.

## 1. Introduction

Newly emerging and reemerging infectious diseases (EID) have been and will continue to be a global threat to transfusion safety. Many notable outbreaks have appeared throughout history: from documented ancient pestilences, through the Middle Ages (*Yersinia pestis*, aka Black Death), and into the 20th century (Influenza, “Spanish Flu”, HIV/AIDS) [1,2]. Increases in global traffic and urbanization, increasingly close animal–human interactions, and climate change all amplify these threats, as newly emerged infectious diseases, such as HIV/AIDS, can spread more widely, and reemerging infectious diseases, such as West Nile virus (WNV) and Zika virus (ZIKV), can more effectively spread to new, naïve populations [3]. One issue stemming from the emergence/reemergence of infectious diseases is the threat they may pose to the blood supply and the increased risk of spread through transfusion-transmitted infections (TTI). Although the risk for TTI due to contaminating pathogens in blood products has been lowered due to advancements in donor history screening as well as pathogen testing, the identification of a new threat and the subsequent development and implementation of an efficient testing protocol is inherently reactive, and may be neither timely nor cost effective [4].

The emergence of severe acute respiratory syndrome coronavirus-2 (SARS-CoV-2) is the latest example of the speed and ease of which an EID can spread worldwide. In December of 2019, a rapidly spreading, new respiratory infection of unknown origin was reported in the Wuhan region in China [5]. The disease, COVID-19, was subsequently shown to be a result of infection with SARS-CoV-2, a betacoronavirus related to, but distinct from, two other significant human pathogens: SARS-CoV and Middle East respiratory syndrome coronavirus (MERS-CoV). In March 2020, a mere three months following its identification, WHO declared COVID-19 a global pandemic [6] and, to date (2 years following its emergence), more than 348 million cases and 5.59 million deaths, and counting, have been reported worldwide.

Although the route of transmission for SARS-CoV-2 was very quickly determined to be via respiratory droplets, the impact on the blood supply and the risk of TTI was not immediately clear. During the SARS-CoV and MERS-CoV outbreaks, multiple studies reported the detection of viral RNA in plasma, serum, and lymphocytes of infected patients, suggesting a theoretical risk of TTI for both viruses [7]. Similarly, low levels of SARS-CoV-2 RNA have been detected in the blood of infected patients, although the correlation between viremia and the severity of disease varies from study to study. For example, in a study on 18 symptomatic and asymptomatic patients in Germany, no SARS-CoV-2 genomes were detected in asymptomatic patients, and, among the symptomatic patients, viremia was detected in only the most severe case [8], suggesting a correlation between viremia and disease severity. This correlation was supported by Chen et al. who observed a link between the detection of viral RNA in the blood and the severity of the disease [9]. In contrast, other studies performed on hospitalized patients reported no correlation between the level of SARS-CoV-2 genomes detected in patients and the presentation of severe disease [10,11]. Furthermore, screening of blood donations has identified SARS-CoV-2 RNA-positive donations from patients who were asymptomatic, although the overall prevalence was low [12,13,14,15]. Although a correlation between viremia versus disease is not well defined, it is clear that SARS-CoV-2 can be found in blood; however, the low prevalence, the inability to isolate infectious virus from SARS-CoV-2 RNA-positive blood samples [16], and, so far, the lack of COVID-19 symptoms in patients that received blood from SARS-CoV-2 RNA-positive donors [12,13], suggests that the risk of SARS-CoV-2 TTI is relatively low. However, during this pandemic, the emergence of SARS-CoV-2 variants has demonstrated the ability of the virus to evolve to improve viral replication, transmission, and immune evasion [17]. Although mixed outcomes have been observed for pathogenicity [17], the continued emergence of variants could affect future TTI risk in ways that are yet unknown. Indeed, although the current risk for TTI is low, new SARS-CoV-2 variants could develop that cause higher viremia in infected patients, which could increase the TTI risk.

**Figure 1:**
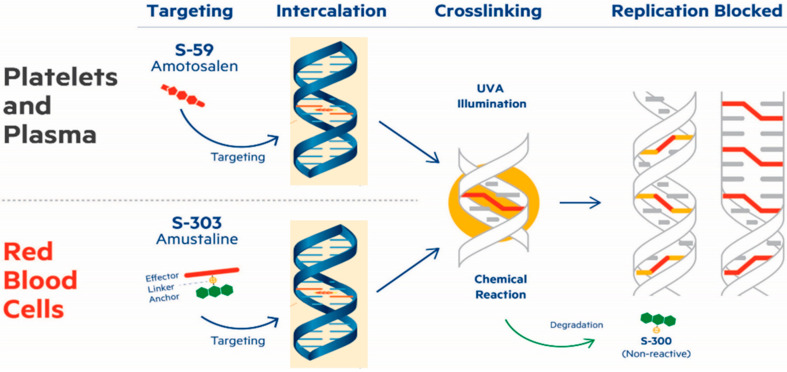
Mechanism of action for amotosalen/UVA and amustaline/GSH. In platelets and plasma (**top**), the amotosalen intercalates into nucleic acids. Treatment with UVA forms irreversible adducts and crosslinks, blocking replication. In red blood cells (**bottom**), the amustaline intercalates into nucleic acids. A rapid chemical reaction forms irreversible adducts and crosslinks, blocking replication, and degradation of amustaline to levels below quantification.

Pathogen reduction technology (PRT) could help mitigate the theoretical risk of SARS-CoV-2 TTI and future unknown threats. The INTERCEPT^®^ Blood System for Platelets and Plasma, which is currently approved and in routine use in many parts of the world, utilizes amotosalen and ultraviolet A light (A/UVA) to inactivate a broad range of viruses, protozoa, and bacteria [18]. This PRT is based on the photochemical treatment of plasma and platelet concentrates with amotosalen, an intercalating agent, to form adducts and irreversible crosslinks within nucleic acids upon UVA illumination, preventing replication, transcription, and translation of contaminating pathogens and leukocytes (Figure 1). The INTERCEPT^®^ Blood System for Red Blood Cells, which is under clinical development, utilizes amustaline (S-303) and glutathione (GSH) for the treatment of red blood cells (RBC). Akin to A/UVA mode of action, treatment with amustaline irreversibly crosslinks nucleic acids, but is not dependent on UVA light (Figure 1). S-303/GSH treatment has been shown to be effective at inactivating several viruses and protozoa, including chikungunya virus (CHIKV) [19,20], ZIKV [21], dengue virus (DENV) [22], and *Plasmodium falciparum* [23]. Previous studies have demonstrated that A/UVA treatment inactivates SARS-CoV and MERS-CoV, two relatives of SARS-CoV-2, in plasma and platelet concentrates (PC) [24,25,26,27]. Most recently, the A/UVA treatment was shown to inactivate SARS-CoV-2 (SARS-CoV-2/human/SAU/85791C/2020) in plasma and PC in plasma [28,29]. This study expands on these initial experiments by investigating the efficacy of the A/UVA PRT to inactivate a second strain of SARS-CoV-2 (USA-WA1/2020) in PC resuspended in 35% plasma/65% platelet additive solution (PC-PAS) in addition to PC resuspended in 100% plasma (PC-100) and plasma. This study also examined the efficacy of the S-303/GSH PRT to inactivate SARS-CoV-2 in RBC, representing the full breadth of transfused blood products. Additionally, the assay system used to detect infectious SARS-CoV-2 in the presence of blood component was validated, ensuring the accuracy and reliability of the results from this and previous studies.

## 2. Results

### 2.1. Validation of SARS-CoV-2 Plaque Assay in Plasma, PC, and RBC

**Figure 2:**
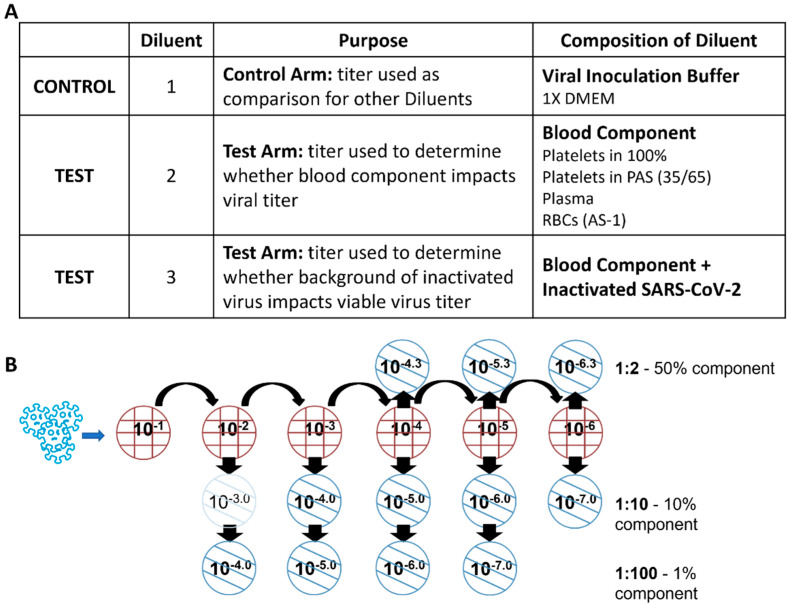
Validation of SARS-CoV-2 plaque assays. (**A**) description of the diluents used in the validation of the SARS-CoV-2 plaque assays; (**B**) schematic representation of the dilution scheme used for the validation of the SARS-CoV-2 plaque assays.

Validation studies were conducted prior to the start of the inactivation experiments to ensure that the presence of the blood component and/or inactivated virions did not affect the ability to detect and enumerate infectious SARS-CoV-2. Diluent 2 and Diluent 3 were used to evaluate the impact of blood component on viral titer and determine the dilution that yields viral titers that are consistent with results from titrations in Diluent 1 (Figure 2). The titers were compared between the diluents to determine the effect of the blood component and inactivated virions on SARS-CoV-2 titers, and to determine the minimum dilution appropriate for the accurate detection of SARS-CoV-2 in test and control samples from inactivation experiments.

Titers in viral inoculation buffer were 5.4 ± 0.1, 5.9 ± 0.0, and 5.5 ± 0.1 log PFU/mL (plaque forming units per mL) for PC-100, PC-PAS, and plasma, respectively. The corresponding titers in Diluent 2 (with 50% blood component) were 5.4 ± 0.0, 5.7 ± 0.0, and 5.5 ± 0.1 log PFU/mL for PC-100, PC-PAS, and plasma, respectively, indicating that the presence of the blood component did not have an impact on the ability to detect infectious SARS-CoV-2 (Table 1). Similar results were obtained with inoculum containing 10% and 1% PC-100, PC-PAS, or plasma and for all titrations performed in Diluent 3 (Table 1).

For AS-1 RBC (also containing processing solution and GSH), the titer in viral inoculation buffer was 5.5 ± 0.1 log PFU/mL and the corresponding titer in Diluent 2 (with 50% AS-1 RBC) was 6.1 ± 0.1 log PFU/mL (Table 1). Although the titer in Diluent 2 was slightly higher compared to Diluent 1, the presence of the AS-1 RBC did not have a negative impact on the ability to detect infectious SARS-CoV-2. Similar results were obtained with inoculum containing 10% and 1% AS-1 RBC and for all titrations performed in Diluent 3 (Table 1).

### 2.2. Inactivation of SARS-CoV-2 in PC-100, PC-PAS, and Plasma

To evaluate SARS-CoV-2 inactivation in platelets, four PC-100 units (1–4) and four PC-PAS units (1–4) were collected, spiked with SARS-CoV-2, and treated with amotosalen (approximately 150 µM) and UVA. For PC-100, the viral titers in the pre-illumination control samples averaged 3.5 ± 0.3 log PFU/mL. No residual virus was detected in the post-illumination test samples, resulting in a mean log reduction factor (LRF) of >3.5 ± 0.3 log PFU/mL (Table 2).

For PC-PAS, the viral titers in the pre-illumination control samples averaged 3.2 ± 0.1 log PFU/mL. No residual virus was detected in the post-illumination test samples, resulting in a mean LRF of >3.2 ± 0.1 log PFU/mL (Table 3). The pre-illumination control titers for both PC-100 and PC-PAS were in the range of the expected values based on the titer of the stock virus (mean of 5.5 ± 0.2 log PFU/mL for PC-100 and mean of 5.2 ± 0.0 log PFU/mL), indicating that neither amotosalen alone nor the blood product alone contributed to the inactivation of SARS-CoV-2.

To evaluate SARS-CoV-2 inactivation in plasma, four plasma pools at approximately 585 mL each were produced from two ABO-matched fresh-frozen plasma components (1–4), spiked with SARS-CoV-2, and treated with amotosalen (approximately 150 µM) and UVA light. The viral titers in the pre-illumination control samples averaged 3.3 ± 0.1 log PFU/mL. No residual virus was detected in the post-illumination test samples, resulting in a mean LRF of >3.3 ± 0.1 log PFU/mL (Table 4).

### 2.3. Inactivation of SARS-CoV-2 in RBC

To evaluate SARS-CoV-2 inactivation in RBC prepared in AS-1 additive solution, four AS-1 RBC units were produced from one to two ABO-matched, leukocyte-reduced whole blood units (1–4), spiked with SARS-CoV-2, and treated with amustaline (approximately 0.17 mM) and GSH (approximately 17 mM). The averaged viral titer in the pre-treatment control sample UT = 0 was 4.2 ± 0.1 log PFU/mL (Table 5). Following the 24-h incubation of each unit at room temperature and subsequent exchange step, no residual virus was detected, resulting in a mean LRF of >4.2 ± 0.1 log PFU/mL. Interestingly, incubation of the pre-treatment control samples for 24 h at room temperature resulted in an approximate 1 log reduction in titer (mean UT = 24 h titer of 3.1 ± 0.1 log PFU/mL compared to the mean UT = 0 titer of 4.2 ± 0.1 log PFU/mL). This loss in infectivity was even more pronounced following the storage of the pre-treatment control sample (UT = 35 d) at 4 °C, with the titers of the units ranging from 0 to only 2.2 log PFU/mL (mean of 1.2 ± 1.1 log PFU/mL).

## 3. Discussion

At the beginning of this pandemic and following the identification of SARS-CoV-2 as the causative agent, the risk of transfusion transmission was unclear, which resulted in changes in deferment policies for donors at blood centers to help mitigate any potential risk. This type of reactionary response can result in dramatic decreases in the blood supply, which puts hospitals in a precarious situation, particularly during a pandemic. This study aims to defuse the reactionary response by using PRT as a potential proactive response to the current pandemic, as well as any other future epidemics or pandemics, to avert potential TTI risks. Additionally, the infectivity assay was validated for PC-100, PC-PAS, plasma, and RBC, ensuring the reliability and accuracy for detecting SARS-CoV-2 in each blood component.

This study follows up previous reports supporting the use of PRT as a viable mitigation against potential TTI [28,29,30,31] by confirming that amotosalen/UVA treatment of PC-100 and plasma inactivates an additional SARS-CoV-2 isolate and provides new evidence that the same technology is also effective in PC-PAS and RBC components. Inactivation to the limit of detection was achieved, resulting in LRF of >3.5 ± 0.3 log PFU/mL in PC-100, >3.2 ± 0.1 log PFU/mL in PC-PAS, and >3.3 ± 0.1 log PFU/mL in plasma. This study also demonstrated robust inactivation of SARS-CoV-2 in AS-1 RBC using S-303/GSH. SARS-CoV-2 was inactivated to the limit of detection, resulting in an LRF of >4.2 ± 0.1 log PFU/mL. As with A/UVA, the fact that no residual virus was detected following treatment indicates that the limits of SARS-CoV-2 inactivation using S-303/GSH have not been reached and, thus, a greater LRF may be achievable. This study represents the first report of complete inactivation of SARS-CoV-2 in RBC preparations. Limited inactivation has been reported (LRF = 3.30 ± 0.26 log PFU/mL, with incomplete inactivation) using riboflavin/UVB in whole blood preparations [31].

Interestingly, when investigating inactivation efficacy in RBCs, there was a pronounced loss in viral titer in the control samples (UT = 24 h and UT = 35 d) following the two incubation steps (mean UT = 24 h titer of 3.1 ± 0.1 log PFU/mL and mean UT = 35 d titer of 1.2 ± 1.1 log PFU/mL). This loss in titer could be caused by a variety of factors: the control samples used in this study contained RBCs plus processing solution and GSH, which could have unknown effects on SARS-CoV-2 stability, or the prolonged storage at 4 °C could have negative effects on SARS-CoV-2 infectivity. Additionally, the control samples in this study were collected and stored side-by-side with the treated RBC unit in 2 mL screw cap cryovials, not an RBC storage container, which could have additional and independent effects on SARS-CoV-2 infectivity. Despite this observation, it is not practical nor safe to delay transfusion of collected units while waiting for any potential contaminating virus to lose infectivity, so PRT remains an important mitigation strategy for reducing the risk of TTI.

The complete, but low level of inactivation observed, is likely a direct result of limitations in the titer of the viral stocks that had been prepared. Furthermore, the input titer is further limited due to spiking with approximately 1% of viral inoculum, based on the volume of the final product (for example, 285 mL platelets + 15 mL amotosalen + 3 mL stock virus), as to not affect the overall composition of each component. Inactivation of the input virus to the limit of detection suggests that the capacity of the system to inactivate SARS-CoV-2 was not reached and a greater LRF could be achieved if a higher viral input titer was used. However, although LRFs are below 4 log PFU/mL (with the exception of the inactivation in RBC), it is expected that PRT may still provide a sufficient proactive protection. This is supported by data that indicates that, to date, only very low levels (high threshold values and/or close to the limit of detection) of SARS-CoV-2 viral RNA have been detected in infected patients and blood donors [9,10,12] and, at the reported RNA levels, infectious virus could not be isolated by cell culture [16]. The low levels of RNAemia observed in infected patients suggests that SARS-CoV-2 do not produce high levels of viremia, so the SARS-CoV-2 titers used in this study may represent a viral titer higher than what would be observed in patients, suggesting that the LRFs presented here may provide sufficient protection from any potential TTI. Additionally, the complete inactivation observed in this study suggests that the capacity of the system to inactivate SARS-CoV-2 was not reached and a greater LRF could be achieved if a higher input viral titer was used. This hypothesis is supported by the fact that higher LRFs were, in fact, achieved for the related SARS-CoV virus, for which higher input virus titers were available, as A/UVA treatment in PC and plasma reported LRFs of >6.2 ± 0.7 log PFU/mL [26] and ≥5.5 ± 0.1 log PFU/mL [27], respectively (Table 6). Additionally, A/UVA treatment effectively inactivated the more distantly related MERS-CoV virus, with LRFs >4.48 log PFU/mL [24] and >4.67 log PFU/mL [25] in PC and plasma, respectively (Table 6). Recently, we also reported efficient inactivation of a different isolate (SARS-CoV-2/human/SAU/85791C/2020) of SARS-CoV-2 in both PC-100 (>3.31 log PFU/mL) [29] and plasma (>3.32 log PFU/mL) (Table 6) [28]. The ability of SARS-CoV-2 to mutate has resulted in the development of multiple variants which, although still related to each other, have affected SARS-CoV-2 infectivity, replication, etc. in different ways. The data presented here, using SARS-CoV-2 isolate USA-WA1/2020, suggest that the sensitivity to A/UVA is not genus or strain-specific; thus, this technology may be effective for any past, presently circulating, or future emergences of members of the *betacoronavirus* genus, including any of the currently circulating SARS-CoV-2 strains/variants.

Recently, the inactivation of SARS-CoV-2 with riboflavin/UVB in single donor plasma and PC was reported. Similar to our studies, riboflavin/UVB treatment also inactivated SARS-CoV-2 to the limit of detection, demonstrating a maximum inactivation of >4.53 log PFU/mL in PC-100 [30], >4.79 log PFU/mL in plasma [31]. The differences in reported inactivation levels are likely attributed to the different input titers, as the reported studies spike units with up to 5% stock virus in the final product, while this study spiked units with only 1% viral stock (to minimize any impact of added volume on the blood component). Thus, the differences in the reported inactivation do not necessarily represent the limitations of each of the treatment methods.

The results for this study support the use of A/UVA PRT for platelets and plasma and S-303/GSH for RBC to mitigate TTI risk during this EID outbreak. While SARS-CoV-2 has not yet been shown to be transfusion-transmitted, its emergence adversely impacted blood availability and revealed an urgent need to address blood continuity as part of preparedness planning. New infectious agents have emerged over the past decade and new ones will continue to emerge in the future. Among those, a new transfusion-transmissible agent may arise. PRT should be considered as an option to maintain blood safety and continuity during EID outbreaks.

Indeed, A/UVA has been used to reduce risk of TTI and availability of blood components in previous outbreaks. A/UVA PRT was implemented for PC in La Réunion, France, during the CHIKV outbreak that began in 2005, to mitigate the risk of TTI and to preserve the blood supply [32]. During the outbreak, in which more than 30% of the population was infected, no CHIKV TTIs were reported. During the ZIKV outbreak in French Polynesia in 2014, A/UVA PRT, implemented in 2010 to control the risk of DENV TTI, allowed for the French territory to maintain the platelet supply. PRT may have prevented possible ZIKV TTI through the transfusion of contaminated PC that underwent PR treatment before transfusion, as suggested by retrospective detection of ZIKV RNA-positive donations [33,34].

Data presented in this study indicate that implementation of A/UVA and, when it becomes available, S-303/GSH PRT, could mitigate risks associated with SARS-CoV-2 TTI during the ongoing pandemic, as well as any future outbreaks caused by agents that have been demonstrated to be susceptible to pathogen inactivation treatment. In fact, broad implementation of A/UVA may be an effective strategy for preemptively protecting the blood supply from both known and unknown threats, as recommended by a European Centers for Disease Control and Prevention (ECDC) expert panel proposing its strategic implementation in areas most at risk for EID emergences [35].

## 4. Materials and Methods

### 4.1. Cell Line and Virus

African green monkey kidney Vero E6 cells (ATCC No. CRL-1586) were maintained in Dulbecco’s Modified Eagle Medium (DMEM) with high glucose (4.5 g/mL) (Thermo Fisher, Waltham, MA, USA) supplemented with 10% fetal bovine serum (FBS), 10% tryptose phosphate broth (TPB), 2 mM L-glutamine, 50 U/mL penicillin, and 50 µg/mL streptomycin (DMEM growth medium).

The SARS-CoV-2 isolate USA-WA1/2020 was obtained from BEI Resources, NIAID, NIH (NR-52281; gene bank accession number: MN985325). The strain was previously isolated in January 2020 from a symptomatic patient who returned to Washington, USA from the impacted region in China. At BEI, the strain was passaged three times on Vero cell cultures and one time on Vero E6 cell cultures. The stock virus used for these studies was prepared by infecting confluent monolayers of Vero E6 cells. Virus was propagated in DMEM growth medium, harvested at 3 days post infection, aliquoted, and stored at −80 °C. All experiments with SARS-CoV-2 were performed in the Biosafety Level 3 facility at the Biosecurity Research Institute (BRI) at Kansas State University (Manhattan, KS, USA).

### 4.2. Platelet, Plasma, and Red Blood Cell Preparation

Apheresis platelet concentrates (PC) suspended in 100% autologous plasma (PC-100) were collected from volunteer donors at Vitalant Research Institute (VRI; Denver, CO, USA) using the Trima^®^ cell separator (Terumo BCT, Lakewood, CO, USA). Apheresis PC suspended in 35% autologous plasma and 65% InterSol^®^ platelet additive solution (PAS; Fenwal Inc., Lake Zurich, IL, USA) (PC-PAS) were collected from volunteer donors at VRI (Denver, CO, USA) using the Amicus^®^ cell separator (Fenwal Inc., Lake Zurich, IL, USA). Each platelet unit was collected in acid citrate dextrose anticoagulant according to AABB (American Association of Blood Banks) standards and shipped to BRI. All platelet units were used within one day of collection.

Whole blood-derived plasma was collected by SunCoast Blood Center (Sarasota, FL, USA) according to AABB standards and provided as fresh-frozen plasma. The previously frozen plasma was rapidly thawed at 37 °C and two ABO-matched units were pooled to generate a unit with a volume of approximately 585 mL.

Whole blood units in citrate–phosphate–dextrose (CPD) were collected at VRI (Denver, CO, USA) and processed, at the blood center, using standard procedures to generate leukocyte-reduced RBC in AS-1 additive solution. The leukocyte-reduced AS-1 RBC were shipped to BRI and used within two days of collection.

### 4.3. Preparation of Heat-Inactivated SARS-CoV-2

The inactivated SARS-CoV-2 stock virus, used in assay validation studies, was prepared using heat inactivation. SARS-CoV-2 stock virus was diluted 1:5 in DMEM growth medium and mixed well. The diluted virus was heat inactivated by incubation at 56 °C for approximately 120 min. Virus inactivation was confirmed by plaque assay and the inactivated virus was aliquoted and stored at −80 °C.

### 4.4. Inactivation in Platelets

Four replicate experiments each were performed for PC-100 and PC-PAS. A single PC unit was used for each replicate. The volume of each of the PC units was adjusted to approximately 285 mL, as determined by weight (density is 1.03 g/mL for PC-100 and 1.01 g/mL for PC-PAS). The PC-100 units contained between 3.8 × 10^11^ to 4.0 × 10^11^ platelets and the PC-PAS units contained between 4.4 × 10^11^ to 5.5 × 10^11^ platelets.

Each unit was spiked with SARS-CoV-2 stock virus at a 1:100 dilution (1% of total platelet plus amotosalen volume; approximately 3 mL). The spiked units were subsequently treated with the INTERCEPT Blood System for Platelets using the Small Volume Processing Set (Cerus Corporation, Concord, CA, USA) according to the manufacturer’s instructions. Spiked PC-100 and PC-PAS units, mixed with 15 mL amotosalen solution (3 mM) in the processing set’s illumination container, were subjected to a single target 3.6 J/cm^2^ UVA light treatment. For each replicate experiment, a stock virus sample, a pre-illumination control sample (following the addition of amotosalen, but prior to UVA illumination), and a post-illumination test sample (following INTERCEPT illumination) were collected for analysis by plaque assay. All samples were stored at −80 °C until analysis.

### 4.5. Inactivation in Plasma

Four replicate experiments were performed for plasma. Pools of two ABO-matched, thawed, fresh-frozen plasma units were used for each replicate. The volume of the plasma pool was adjusted to approximately 585 mL, as determined by weight (density is 1.023 g/mL).

Each unit was spiked with SARS-CoV-2 stock virus at a 1:100 dilution (1% of total plasma plus amotosalen volume; approximately 6 mL). The spiked units were treated with the INTERCEPT Blood System for Plasma (Cerus Corporation, Concord, CA, USA) according to the manufacturer’s instructions. Spiked plasma units, mixed with 15 mL amotosalen solution (6 mM) in the set’s illumination container, were subjected to treatment with a single target dose of 6.4 J/cm^2^ UVA light. Samples were collected from each replicate experiment as described above and all samples were stored at −80 °C until analysis.

### 4.6. Inactivation in Red Blood Cells

Four replicate experiments were performed for AS-1 RBC, as previously described [21,22], with some modifications. AS-1 RBC received at BRI were pooled within blood type, if necessary, and adjusted, by weight (density is 1.06 g/mL) to approximately 360 mL. Each unit was spiked with SARS-CoV-2 stock virus at a 1:100 dilution (1% of total AS-1 RBC plus processing solution, GSH, and S-303; approximately 5.3 mL) and treated with the INTERCEPT Blood System for RBC as previously described [19,20], with some modifications. The INTERCEPT Blood System for RBC comprises an S-303 vial, a GSH vial, a trifurcated set with two 0.2-µm filters and a blind lead, and a processing set with three containers: a mixing bag containing a proprietary processing solution, an incubation bag, and a storage bag containing AS-1 additive solution. The trifurcated filter set was sterilely attached to the mixing bag of the processing set and the SARS-CoV-2 contaminated unit was attached to the blind lead. GSH and the contaminated units were added to the mixing bag and mixed to ensure proper homogenization. Three pre-treatment samples were collected from all units: one was frozen immediately after collection (untreated control, UT = 0), along with a sample of the stock virus, and stored at −80 °C until analysis. The other two pre-treatment control samples were incubated along with the treated units. Following sample collection, amustaline was added to the mixing bag and the RBC (containing processing solution, GSH, and amustaline) were transferred to the incubation bag. The unit, alongside the collected control samples, was stored at room temperature for 24 h. After incubation, an exchange step was performed as previously described [19] and a post-treatment test sample (Test, T = 24 h) was collected from each unit. This sample, along with one of the remaining control samples (untreated control, UT = 24 h) was stored at −80 °C until analysis. Each unit was then transferred to 4 °C and stored for a total of 35 days post collection. Following this storage period, a post-storage test sample (Test, T = 35 d) was collected from each unit. This sample, along with the remaining control sample (untreated control, UT = 35 d) was stored at −80 °C until analysis.

### 4.7. Plaque Assay

Plaque assays were performed in Vero E6 cells to determine the titer of infectious virus in all stocks and blood products spiked with SARS-CoV-2. Frozen samples were thawed and diluted in DMEM growth medium supplemented with 5 µg/mL heparin (viral inoculation buffer). Heparin was included in the diluent to prevent the formation of fibrin clots, which can form when the anticoagulant in the blood encounters the divalent cations in the culture medium. These clots are disruptive to the monolayer, which may result in a loss in assay sensitivity. Stock and control samples were serially diluted 10-fold and test samples were diluted 1:2 to avoid toxicity associated with the blood products, which would impair the ability to detect any residual infectious virus. Inocula were added to 6 well plates containing confluent Vero E6 monolayers (1-mL volumes in quadruplicate for stock and control samples and 30 wells for test samples) and allowed to incubate for approximately 1 h, with agitation every 15 min. After incubation, the inocula were removed and all wells were rinsed with Dulbecco’s phosphate buffered solution (DPBS) and overlaid with DMEM growth medium containing 1.5% methylcellulose. After 5 days of incubation at 37 °C in a 5% CO_2_ atmosphere, titers were determined by fixing the plates with formalin and staining with 1% crystal violet solution. Plaques were enumerated for each dilution macroscopically and viral titers were expressed as PFU/mL.

### 4.8. Validation of Plaque Assays

Prior to the start of the inactivation studies, the plaque assay was validated for use in each component (PC-100, PC-PAS, plasma, and AS-1 RBC). This was carried out to ensure that the presence of the blood component and/or inactivated virions in the inoculum did not interfere with the ability to detect viable SARS-CoV-2 when using the Vero E6 plaque assay. To validate the plaque assay, SARS-CoV-2 was titrated in three diluents (Figure 2A). Diluent 1 consisted of viral inoculation buffer (DMEM growth medium containing 5 µg/mL heparin). Diluent 2, which mimicked control samples collected during inactivation experiments, consisted of aliquots of PC (either PC-100 or PC-PAS), plasma, or AS-1 RBC. Diluent 3, which mimicked test samples collected during inactivation experiments, consisted of the same aliquots of PC (either PC-100 or PC-PAS), plasma, or AS-1 RBC containing a background of heat-inactivated SARS-CoV-2. Diluent 3 was generated by diluting the heat-inactivated SARS-CoV-2 1:10 into the blood component being tested.

SARS-CoV-2 stock virus was serially diluted in each of the diluents. For Diluents 2 and 3, sub-dilutions into viral inoculation buffer were prepared at 1:2, 1:10, and 1:100 (Figure 2B). This accounts for inoculum containing 50%, 10%, and 1% of the blood component. The dilution into viral inoculation buffer was necessary to prevent toxicity of the blood component to the Vero E6 monolayer, as well as to determine the appropriate dilution for the test samples during inactivation experiments. The prepared dilutions were then assayed by plaque assay as described above.

## Figures and Tables

**Table 1:** Validation of SARS-CoV-2 plaque assay: infectious titers in viral inoculation buffer, blood component, or blood component with inactivated virions.

	Viral Infectivity Titer (LOG10 PFU/mL) ^a^						
Component		Blood Component (Diluent 2)			Blood Component + Inactivated SARS-CoV-2 (Diluent 3) ^b^		
Inoculum Composition	NA	50%	10%	1%	50%	10%	1%
PLASMA	5.5 ± 0.2	5.5 ± 0.1	5.5 ± 0.1	5.6 ± 0.1	5.5 ± 0.0	5.5 ± 0.0	5.6 ± 0.0
PC (35/65)	5.9 ± 0.0	5.7 ± 0.0	5.7 ± 0.1	5.6 ± 0.1	5.7 ± 0.1	5.8 ± 0.1	5.7 ± 0.1
PC (100%)	5.4 ± 0.1	5.4 ± 0.0	5.2 ± 0.2	5.2 ± 0.1	5.5 ± 0.1	5.3 ± 0.2	5.2 ± 0.2
AS-1 RBC ^c^	5.5 ± 0.1	6.1 ± 0.0	5.3 ± 0.3	5.0 ± 0.2	6.0 ± 0.2	5.2 ± 0.2	5.1 ± 0.2

^a^ Titers represent mean and standard deviation of three independent experiments. ^b^ Diluent 3 contained approximately 4–5 log PFU/mL of heat-inactivated SARS-CoV-2. Complete inactivation was confirmed prior to the start of the validation. ^c^ Contains processing solution and GSH.

**Table 2:** Infectious titers of SARS-CoV-2 in platelet concentrates prepared in 100% plasma before and after treatment with amotosalen/UVA.

	Viral Infectivity Titer (LOG10 PFU/mL)			
Unit	Stock	Pre-Illumination *	Post-Illumination	Log Reduction Factor
1	5.7	3.9	ND	>3.9
2	5.4	3.3	ND	>3.3
3	5.4	3.4	ND	>3.4
4	5.6	3.4	ND	>3.4
Mean ± SD	5.5 ± 0.2	3.5 ± 0.3	ND	>3.5 ± 0.3 ^¥^

ND = not detected/no plaques detected at dilutions tested. * After addition of amotosalen. ^¥^ Designates inactivation to the limit of detection for all replicates.

**Table 3:** Infectious titers of SARS-CoV-2 in platelet concentrates prepared in 35% plasma/65% PAS before and after treatment with amotosalen/UVA.

	Viral Infectivity Titer (LOG10 PFU/mL)			
Unit	Stock	Pre-Illumination *	Post-Illumination	Log Reduction Factor
1	5.2	3.2	ND	>3.2
2	5.2	3.3	ND	>3.3
3	5.1	3.2	ND	>3.2
4	5.2	3.2	ND	>3.2
Mean ± SD	5.2 ± 0.0	3.2 ± 0.1	ND	>3.2 ± 0.1 ^¥^

ND = not detected/no plaques detected at dilutions tested. * After addition of amotosalen. ^¥^ Designates inactivation to the limit of detection for all replicates.

**Table 4:** Infectious titers of SARS-CoV-2 in human plasma before and after treatment with amotosalen/UVA.

	Viral Infectivity Titer (LOG10 PFU/mL)			
Unit	Stock	Pre-Illumination *	Post-Illumination	Log Reduction Factor
1	5.5	3.3	ND	>3.3
2	5.7	3.3	ND	>3.3
3	5.4	3.4	ND	>3.4
4	5.5	3.4	ND	>3.4
Mean ± SD	5.5 ± 0.1	3.3 ± 0.1	ND	>3.3 ± 0.1 ^¥^

ND = not detected/no plaques detected at dilutions tested. * After addition of amotosalen. ^¥^ Designates inactivation to the limit of detection for all replicates.

**Table 5:** SARS-CoV-2 quantitation in RBC before and after treatment with amustaline/GSH.

	Viral Infectivity Titer (LOG10 PFU/mL)						
		Pre-Treatment Samples *			Post-Treatment Samples		
Unit	Stock	UT = 0	UT = 24 h	UT = 35 d	T = 24 h	T = 35 d	Log Reduction Factor
1	6.2	4.1	3.0	2.2	ND	ND	>4.1
2	5.3	4.1	3.1	ND	ND	ND	>4.1
3	6.2	4.3	3.2	2.1	ND	ND	>4.3
4	6.1	4.1	3.0	0.4	ND	ND	>4.1
Mean ± SD	6.2 ± 0.1	4.2 ± 0.1	3.1 ± 0.1	1.2 ± 1.1	ND	ND	>4.2 ± 0.1 ^¥^

ND = not detected/no plaques detected at dilutions tested. * After addition of processing solution and GSH. ^¥^ Designates inactivation to the limit of detection for all replicates.

**Table 6:** Comparison of log reduction factors for past and present *betacoronaviruses* using amotosalen/UVA and amustaline/GSH.

	Blood Component			
Pathogen	PC-PAS	PC-100	Plasma	RBC
SARS-CoV-2 (USA-WA1/2020)	>3.2 ± 0.1	>3.5 ± 0.3	>3.3 ± 0.1	>4.2 ± 0.1
SARS-CoV-2 (SARS-CoV-2/HUMAN/SAU/85791C/2020)	nt	>3.31 ± 0.23 [29]	>3.32 ± 0.2 [28]	nt
SARS-CoV	>6.2 ± 0.7 [26]	nt	≥5.5 ± 0.1 [27]	nt
MERS-CoV	nt	>4.48 ± 0.3 [24]	>4.67 ± 0.25 [25]	nt

nt = not tested.

## Data Availability

The datasets supporting the findings of this article are included in the article. Any data summarized in this study are available from the corresponding author upon reasonable request.
